# Evaluation of novel radiation protection devices during radiologically guided interventions

**DOI:** 10.1186/s42155-024-00430-0

**Published:** 2024-02-14

**Authors:** Maria E.V. Larsson, Pernilla I. Jonasson, Petra S. Apell, Peter P. Kearney, Charlotta J. Lundh

**Affiliations:** 1 Department of Medical Radiation Sciences, Institute of Clinical Sciences, Sahlgrenska Academy at University of Gothenburg, Gothenburg, Sweden; 2 Department of Medical Physics and Biomedical Engineering, Sahlgrenska University Hospital, Gothenburg, Sweden; 3 Texray AB, Gothenburg, Sweden; 4 Department of Technology Management and Economics, Chalmers University of Technology, Gothenburg, Sweden; 5 Department of Cardiology, Cork University Hospital, Cork, Ireland

**Keywords:** Radiation exposure, Ionizing radiation, Scatter radiation, Radiation protection, Personal protection equipment, PPE

## Abstract

**Background:**

In radiologically guided interventions, medical practitioners are subjected to radiation exposure, which may lead to radiation-induced diseases. In this study, novel radiation shields for the head and neck were evaluated for their potential to reduce radiation exposure.

**Method:**

An anthropomorphic phantom was exposed on its left side to scattered radiation from beneath to simulate the exposure of an operator in a x-ray operating room. Thermoluminescent dosimeters (TLDs) were positioned at different depths in five slices in the phantom, measuring personal dose equivalent. Two different set up situations were evaluated: a head protector designed to reduce radiation in the upper section of the head; and a novel thyroid protector prototype extended in the front and on both sides, designed to reduce radiation in the lower and middle sections of the head. A standard thyroid collar prototype and a ceiling mounted lead glass shield were used as comparisons.

Furthermore, the head protector was evaluated in a clinical study in which TLDs were positioned to measure scattered radiation exposure to the heads of operators during endovascular interventions.

**Results:**

The extended thyroid protector reduced the scattered radiation in the throat, chin, and ear slices. Some shielding effect was seen in the brain and skull slices. The head protector showed a shielding effect in the skull slice up to two cm depth where it covered the phantom head. As expected, the ceiling mounted lead glass shield reduced the scattered radiation in all measuring points.

**Conclusions:**

A ceiling mounted lead glass shield is an effective radiation protection for the head, but in clinical practice, optimal positioning of a ceiling mounted lead shield may not always be possible, particularly during complex cases when radiation protection may be most relevant. Added protection using these novel guards may compliment the shielding effect of the ceiling mounted lead shield. The head protector stand-alone did not provide sufficient protection of the head. The extended thyroid protector stand-alone provided sufficient protection in the lower and middle sections of the head and neck.

**Graphical Abstract:**

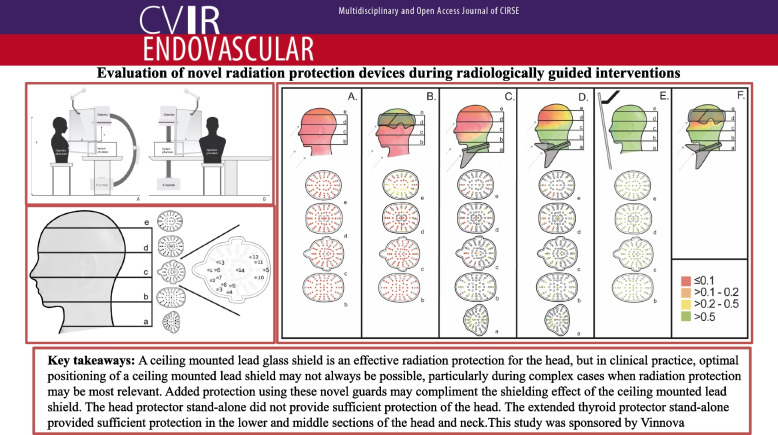

## Background

In radiologically guided interventions, Medical practitioners are subjected to x-ray exposure, which may exceed occupational dose limits and lead to radiation-induced diseases [1].

Regulations for occupational dose limits follow recommendations by the International Commission on Radiological Protection (ICRP). In ICRP 118, the commission considered the radiation effect in the lens of the eye and recommended keeping doses below the nominal threshold 0.5 Gy to prevent radiogenic cataract and they also recommended a reduced occupational dose limit [2]. It has been suggested when wearing protective lead glasses to reduce the exposure to the lens of the eye, a dose reduction factor (DRF) of at least 2 could be applied [3, 4].

According to ICRP 103 the radiation-induced cancer sensitivity of the brain is relatively low [5], and the need for head protection might seem unnecessary. However, the risk of tissue reaction effects are not completely understood [6, 7], and awareness is raised by the commission in ICRP 118 [2] for radiation-induced circulatory diseases suggesting that the radiation dose threshold level for the brain might be 0.5 Gy corresponding to the threshold for the lens of the eye. Furthermore, reports of left-sided brain tumors in interventional cardiologists [8–10], has increased interest among medical practitioners in reducing the radiation exposure of the head.

Many types of radiation protection devices can be used to reduce scattered radiation [11, 12]. Personal protection equipment such as aprons, thyroid collar, and lead eyeglasses, as well as ceiling mounted lead shields, is commonplace in most x-ray operating rooms. The type of radiation protection devices used by personnel depends on the clinical situation. Ceiling mounted lead shields can effectively reduce the dose to the operator. However, the dose-reducing effect of a lead shield is directly correlated to the accuracy of the positioning of the shield [12]. Personal protection equipment has the advantage of keeping the protection relatively constant despite the wearer moving about. Conventional radiation protection aprons do not, however, cover certain body parts such as the head. It is of interest to evaluate head and neck radiation protection devices as they can be valuable in clinical situations involving high radiation exposure, given the practical limitations in achieving adequate protection from ceiling mounted lead glass shields in certain C-arm positions and during certain procedural maneuvers.

The aim of this study was to evaluate two novel radiation protection devices to assess if these shields can reduce the absorbed dose to the head and neck during radiologically guided interventions.

## Methods

**Fig. 1:**
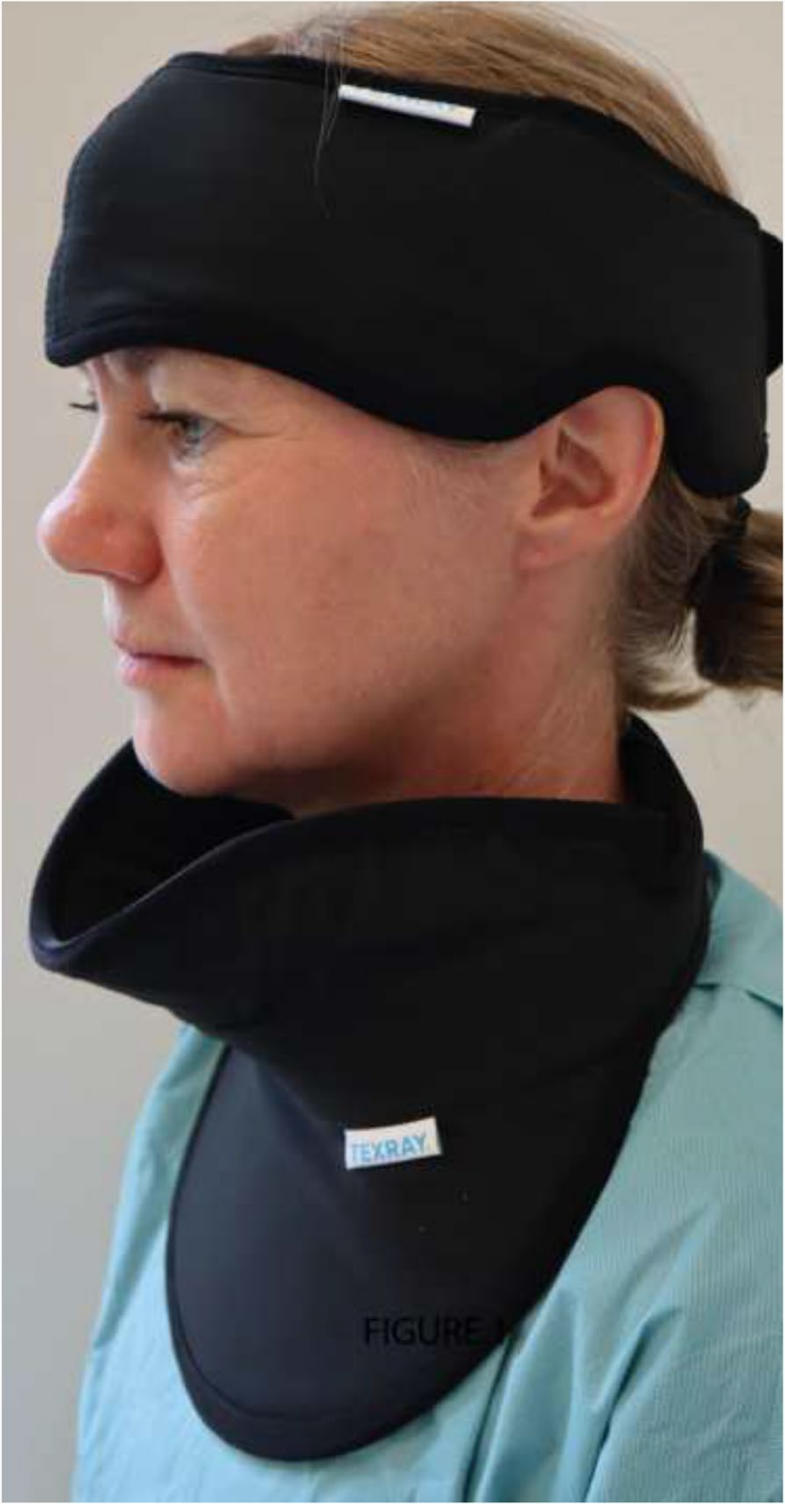
The head protector used in both the phantom study and the clinical study and the extended thyroid protector used in the phantom study

Two different head and neck protection devices were evaluated (Fig. 1). The head protector (HeadPeace™,Texray AB, Sweden), with 0.25 mm lead equivalence, is designed to provide radiation protection in the upper section of the head. The extended thyroid protector (MindPeace™, patent pending, Texray AB, Sweden) evaluated in this study was a prototype of 0.5 mm lead equivalence, extended in the front and on both sides, and designed to provide radiation protection in the lower and middle sections of the head.

Thermoluminescent dosimeters (TLD) DXT-RAD Ringlets TLD-100 (Thermo Fisher Scientific Inc) were used to measure radiation dose at each measuring point. The TLDs are normally used in the Personal Dosimetry Laboratory at Sahlgrenska University hospital and were calibrated for personal dose equivalent Hp (0.07) in N80 spectrum, according to ISO 4037–1,-3, [13].

### Phantom study

**Fig. 2:**
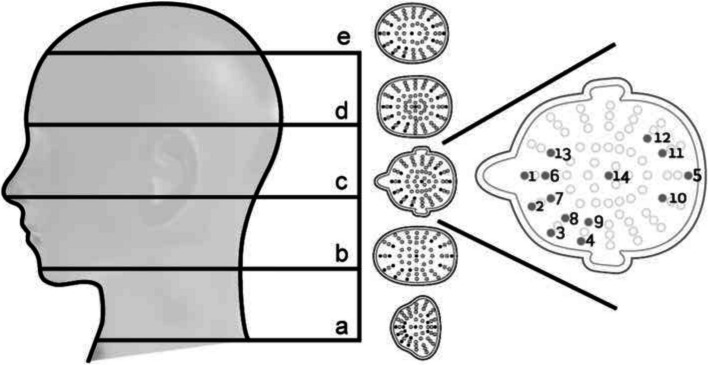
Illustration of the phantom used to simulate the operator’s head where TLDs were positioned at different depths in five slices (**a**, **b**, **c**, **d** and **e**). For the head protector and ceiling mounted lead shield studies all TLD positions (light grey and dark grey) were used, while for the extended thyroid protector study the TLD positions 1–14 (dark grey) were used. On the right, slice c is shown enlarged, illustrating the numbering in each individual section

To study exposure to the head and neck, a study setup was developed to simulate a radiologically guided intervention. An anthropomorphic phantom, head, and thorax, (Rando Alderson, USA) was used to simulate a medical practitioner, exposed to scattered radiation. In the phantom head, TLDs were positioned at different depths in five slices (a, b, c, d and e), as shown in Fig. 2. Each slice was 5 cm apart, and in between each slice a 2 mm high polymethyl methacrylate (PMMA) slice was inserted, drilled with holes for placement of TLDs. In the head protector and ceiling mounted lead shield studies all TLD position were used while for the extended thyroid protector the TLD position 1–14 were used, (Fig. 2). The thorax phantom part, was equipped with a radiation protective apron of 0.5 mm lead equivalence.

**Fig. 3:**
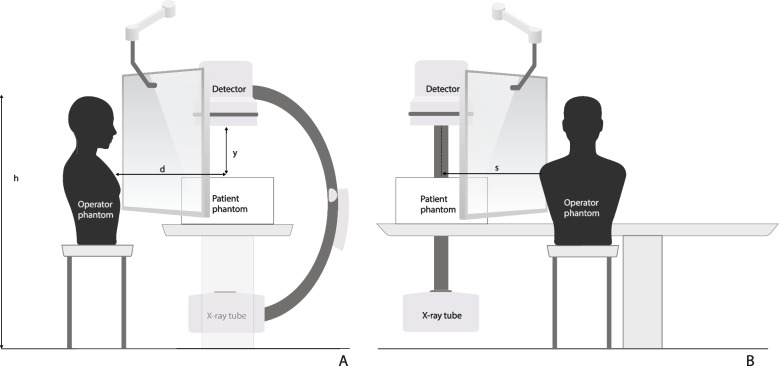
A schematic phantom set-up to illustrate a medical practitioner performing a radiologically image-guided procedure. **A** shows the measurement setup seen from the foot end of the patient phantom and **B** the measurement setup seen from the left side of the patient phantom, See Table 1 for details

Another phantom was placed on a table to simulate a patient undergoing an x-ray examination. The x-ray equipment was positioned with the x-ray tube beneath the table set at zero-degree angle. The operator-phantom was placed facing the table, with the exposed area of the patient-phantom to the left, as shown in Fig. 3, imitating a typical exposure situation during x-ray guided interventions, with scattered radiation hitting the operator on the left side from beneath. During the evaluation of the head protector, four 5 cm PMMA blocks were used to simulate a patient (20 × 30x25 cm) and during the evaluation of the extended thyroid protector, a 21 cm thick anthropomorphic phantom was used. Exposure-parameters are presented in Table 1.

**Table 1:** Exposure-parameters for phantom study

Parameters	Head protector and ceiling mounted lead shield	Extended thyroid protector and standard thyroid collar
**Modality**	Siemens Artis Q^a^	Philips Multi Diagnost Eleva FD^b^
**Table height (cm)**	90	80
**Operator phantom height, h (cm)**	165	165
**Central beam axis—operator phantom front distance, d (cm)**	26	25
**Central beam axis—operator phantom side distance, s (cm)**	58	66
**Protocol**	Abdomen	Abdomen
**Exposure mode**	Acquisition	Acquisition
**Zoom (cm)**	42	48
**Source image distance**	120	125
**Patient phantom surface—detector distance, y (cm)**	50	54
**Frames per second**	4	2
**Tube voltage (kV)**	70	90
**Tube load (mAs)**	30	25
**Filter**	No	1 mm Al, 0.1 mm Cu
**DAP**^**c**^ **(Gycm2)**	400	130

^a^Siemens Artis Q (Siemens Healthineers, Germany) angio system

^b^Philips MultiDiagnost Eleva FD (Philips, The Netherlands)

^c^Dose Area Product, to terminate the radiation at the same exposure-level

#### Evaluation of the head protector and extended thyroid protector

To evaluate the radiation shielding effect of the protectors, a shielding-effect ratio, R_SE_, was developed for every measuring point in the head phantom, comparing the TLD-signal, including the radiation protection, with the TLD-signal without the evaluated radiation protection, where R_SE_ = 1-[Hp(0.07)_shielded_/Hp(0.07)_unshielded_)].

For evaluation of the head protector, the PMMA slices in the head phantom (b-e) were prepared with 224 TLDs (53, 57, 59 and 55 TLDs per slice). As comparison a ceiling mounted lead shield with 0.5 mm lead equivalence was used. Meanwhile for the evaluation of the extended thyroid protector, the PMMA slices in the head phantom (a-e) were prepared with 70 TLDs (14 per slice). As comparison, a prototype of a standard thyroid collar, with 0.5 mm lead equivalence, of the same attenuating material as the extended thyroid protector used in this study, was evaluated. The dose-area-product (DAP) indication on the x-ray equipment was used to terminate the radiation at the same exposure-level between set-ups.

### Clinical study

**Fig. 4:**
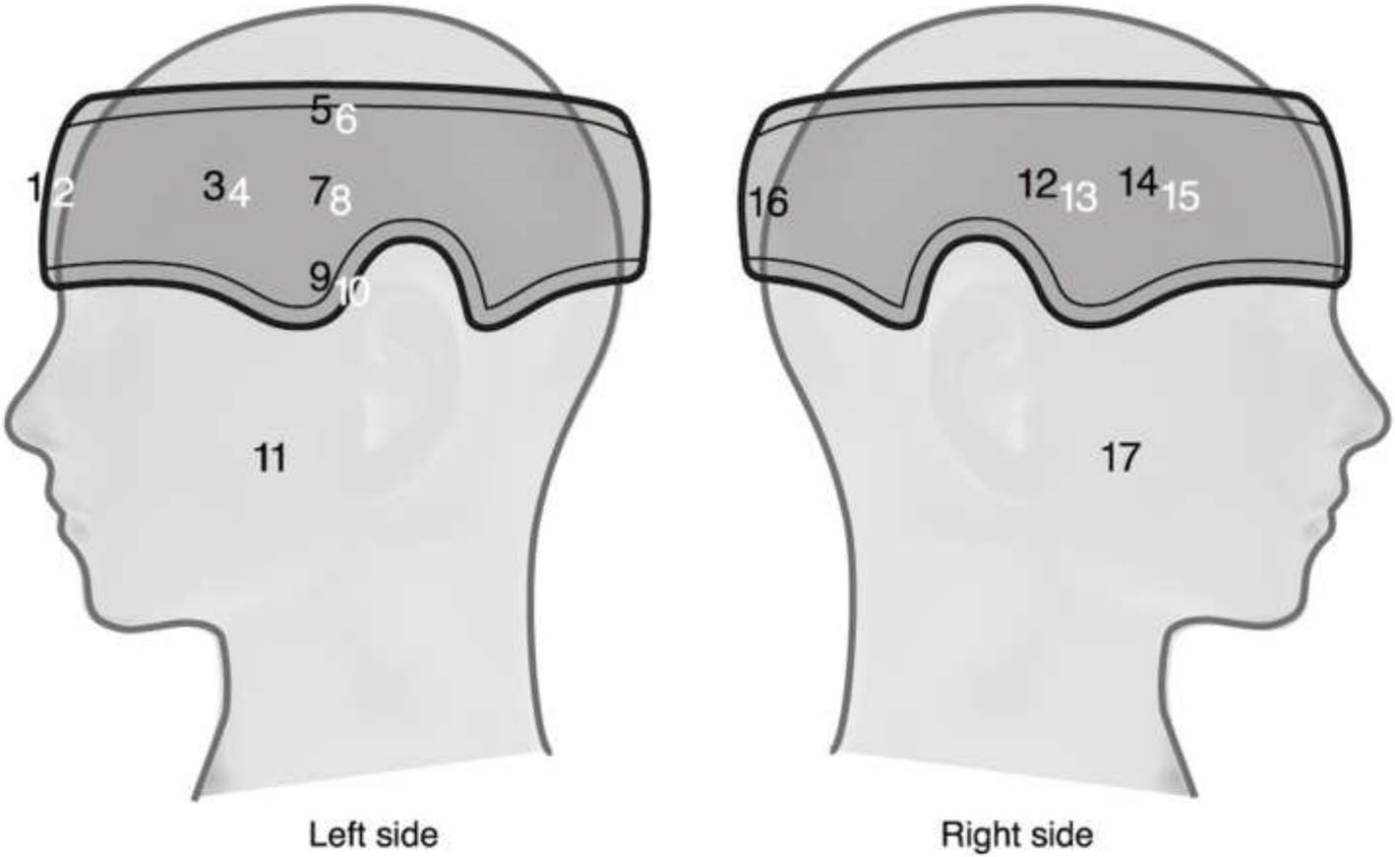
Illustration of the TLD-pocket positions. Positions marked black are on the outside of the head protector and positions marked white are on the inside of the head protector

The clinical study was performed at two sites, Cork University Hospital (Cork, Ireland) and Sahlgrenska University Hospital (Gothenburg, Sweden). Fourteen study participants representing Cardiac, Electrophysiology, Hybrid OR and Gastrointestinal Radiology departments were included. Each participant used the head protector in clinical practice during a period of thirty days. The participants followed usual radiation-protection routines in their respective departments and the head protector was used as complimentary personal protection equipment. TLDs were placed in 16 lining pockets inside and outside of the attenuating material, in a specifically modified head protector to enable evaluation of the shielding effect, (Fig. 4).

To estimate the absolute personal dose equivalent, a background subtraction was performed, using the mean TLD-signal from dosimeters placed in the inside and the outside lining pockets of a separate head protector placed in an office at each department during the measuring period.

The personal dose equivalent was compared between the inside and outside of the head protector to get a shielding effect ratio, i.e. R_SE_ = 1-[Hp(0.07)_shielded_/Hp(0.07)_unshielded_)]. No background subtraction was performed when calculating the shielding effect.

## Results

### Phantom studies

The R_SE_ for every measuring point in the head phantom are presented in Table 2 for the head protector, the extended thyroid protector, the standard thyroid collar and the ceiling mounted lead glass screen, respectively. A R_SE_ of 1 represent an optimal shielding effect, and 0 no shielding effect.

**Table 2:** The shielding effect ratio, R_SE_, from the TLD-measure data Hp (0.07) for slices a-e and each respective position according to Fig. 2

	**Head protector**				**Extended thyroid protector**				
Position/Slice	**b**	**c**	**d**	**e**	**a**	**b**	**c**	**d**	**e**
**1**	0.0^a^	0.0^a^	0.0^a^	0.9	1.0	0.9	0.8	0.2	0.0
**2**	0.0^a^	0.0^a^	0.0^a^	0.9	1.0	0.9	0.7	0.2	0.1
**3**	0.0^a^	0.0^a^	0.0^a^	0.9	0.9	0.9	0.5	0.1	0.2
**4**	0.0	0.0^a^	0.1	0.9	0.9	0.9	0.5	0.1	0.1
**5**	0.0	0.0^a^	0.0	0.1	0.6	0.8	0.6	0.3	0.6
**6**	0.0^a^	0.1	0.0	0.2	1.0	1.0	0.8	0.2	0.2
**7**	0.0^a^	0.0	0.0^a^	0.2	0.9	0.9	0.8	0.2	0.2
**8**	0.0^a^	0.0^a^	0.0^a^	0.4	0.8	1.0	0.8	0.1	0.2
**9**	0.0^a^	0.0^a^	0.0	0.5	0.8	0.9	0.7	0.2	0.2
**10**	0.1	0.0	0.0	0.2	0.6	0.8	0.7	0.5	0.2
**11**	0.0^a^	0.0^a^	0.2	0.1	0.6	0.8	0.7	0.4	0.3
**12**	0.0^a^	0.0^a^	0.0^a^	0.4	0.2	0.9	0.8	0.5	0.4
**13**	0.0^a^	0.0^a^	0.0^a^	0.0	0.9	0.9	0.8	0.5	0.2
**14**	0.0^a^	0.0	0.0^a^	0.1	0.0	0.9	0.9	0.5	0.4

^a^R_SE_ < 0 is set to 0

**Fig. 5:**
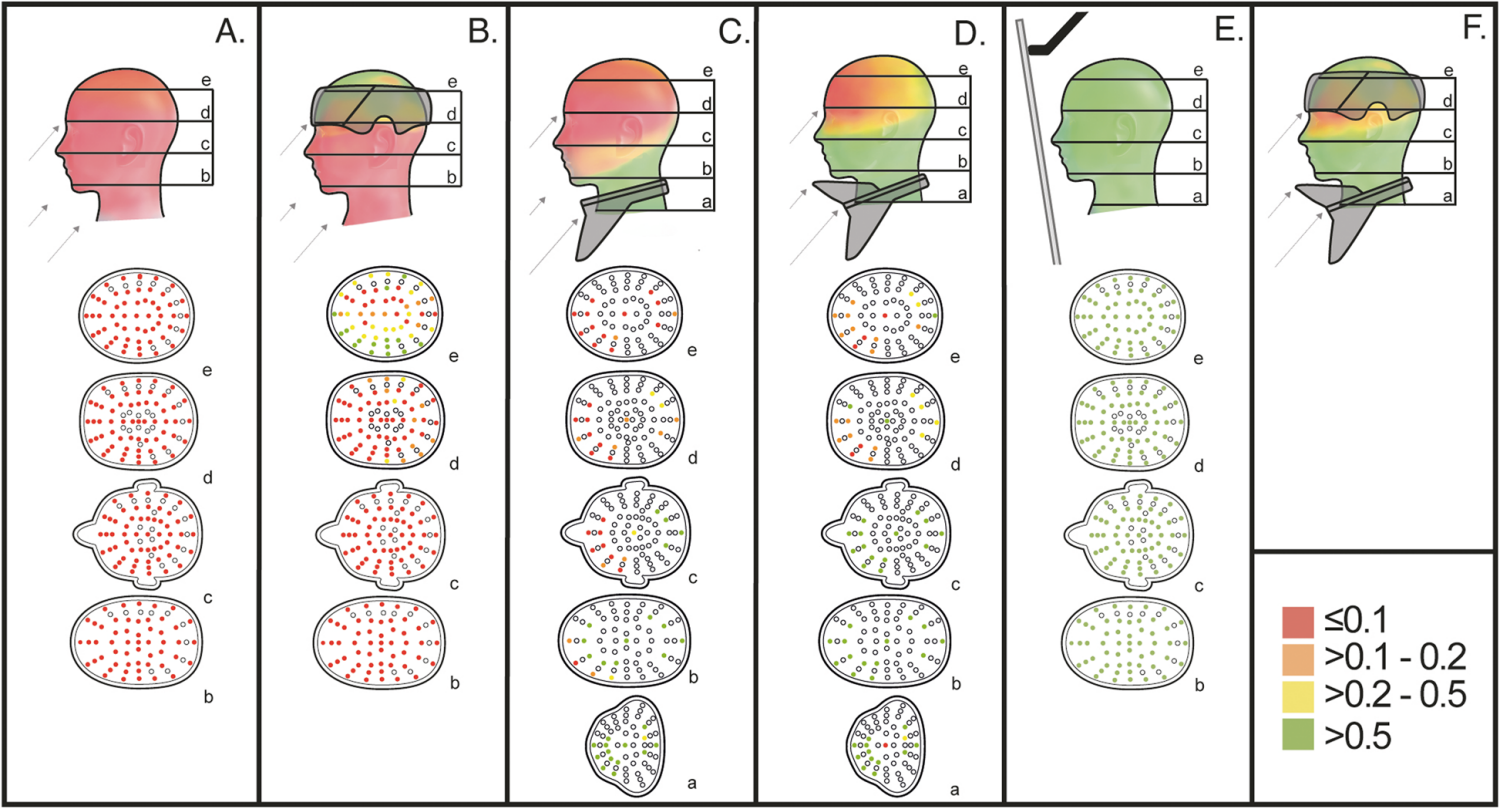
Schematic illustration of the shielding effect, RSE, where green indicates RSE > 0.5; yellow RSE > 0.2–0.5; orange RSE > 0.1–0.2; and red RSE ≤ 0.1. The following set-ups were used **A** no head-protection; **B** the head protector; **C** the prototype standard thyroid collar; **D** the extended thyroid protector; **E** ceiling mounted lead glass shield; and **F** a predicted result from a combination of the head protector and the extended thyroid protector. The shielding-effect ratio for the unshielded set-up A the ratio is: RSE = 1-[Hp(0.07)unshielded/Hp(0.07)unshielded)]. To see the RSE at depth look at respective slice a-e with the coloured measure-positions in each figure A-E

A schematic color illustration of the shielding effect, R_SE_, where green indicates R_SE_ > 0.5; yellow R_SE_ > 0.2–0.5; orange R_SE_ > 0.1–0.2; and red R_SE_ ≤ 0.1, was created for the following set-ups A) no head protection; B) the head protector; C) the standard thyroid collar; D) the extended thyroid protector; and E) the ceiling mounted lead glass shield (Fig. 5). In accordance with the reported dose reduction factor of an average pair of lead glasses [4], 50% was indicated as green in this visual presentation. Between the slice-layers and between measure-point within the layer, a gradual combination of colors was used in the event the shielding effect differed between the different slices.

As a comparison, the shielding-effect ratio for the unshielded set-up is illustrated in Fig. 5A: RSE = 1-[Hp(0.07)_unshielded_/Hp(0.07)_unshielded_)] i.e. RSE = 0 in all measure-points.

For the head protector, a shielding effect was seen on the left side in slice e, meanwhile as expected there was no shielding effect in the uncovered lower slices b-d, see Table 2 and as illustrated in Fig. 5B. For slice e, the mean shielding effect was 0.3 (SD 0.3; range 0 to 0.9) (data not shown). The largest shielding effect was found on the left side of the phantom head where the shielding effect at 0.5 cm depth was 0.9 (slice e, position 1, 2, 3 and 4), but gradually decreased with depth, at 2.5 cm depth the R_SE_ was 0.2 to 0.5 (slice e, position 6, 7, 8 and 9), and at the centre 0.1 (slice e, position 14), see Table 2. For the standard thyroid collar, a shielding effect was seen in slice a-b, whereas no shielding effect was noted in the middle or upper slices c-e, as illustrated in Fig. 5C.

The TLD-data show a shielding effect of the extended thyroid protector in slices a-c with a mean shielding effect of 0.7 (SD 0.3; range 0.0 to 1.0), 0.9 (SD 0.1; range 0.8 to 1.0), and 0.7 (SD 0.1; range 0.5 to 0.9) respectively, see Table 2 and as illustrated in Fig. 5D. A minor shielding effect of the extended thyroid protector was also seen in slices d-e, particularly in the TLD-positions in the central and back part of the head.

The shielding effect of the ceiling mounted lead shields was more than 0.9 in the great majority of measured TLD-positions, as illustrated in Fig. 5E.

A predicted result from a combination of the head protector and the extended thyroid protector is schematically illustrated in Fig. 5F.

### Clinical study

**Fig. 6:**
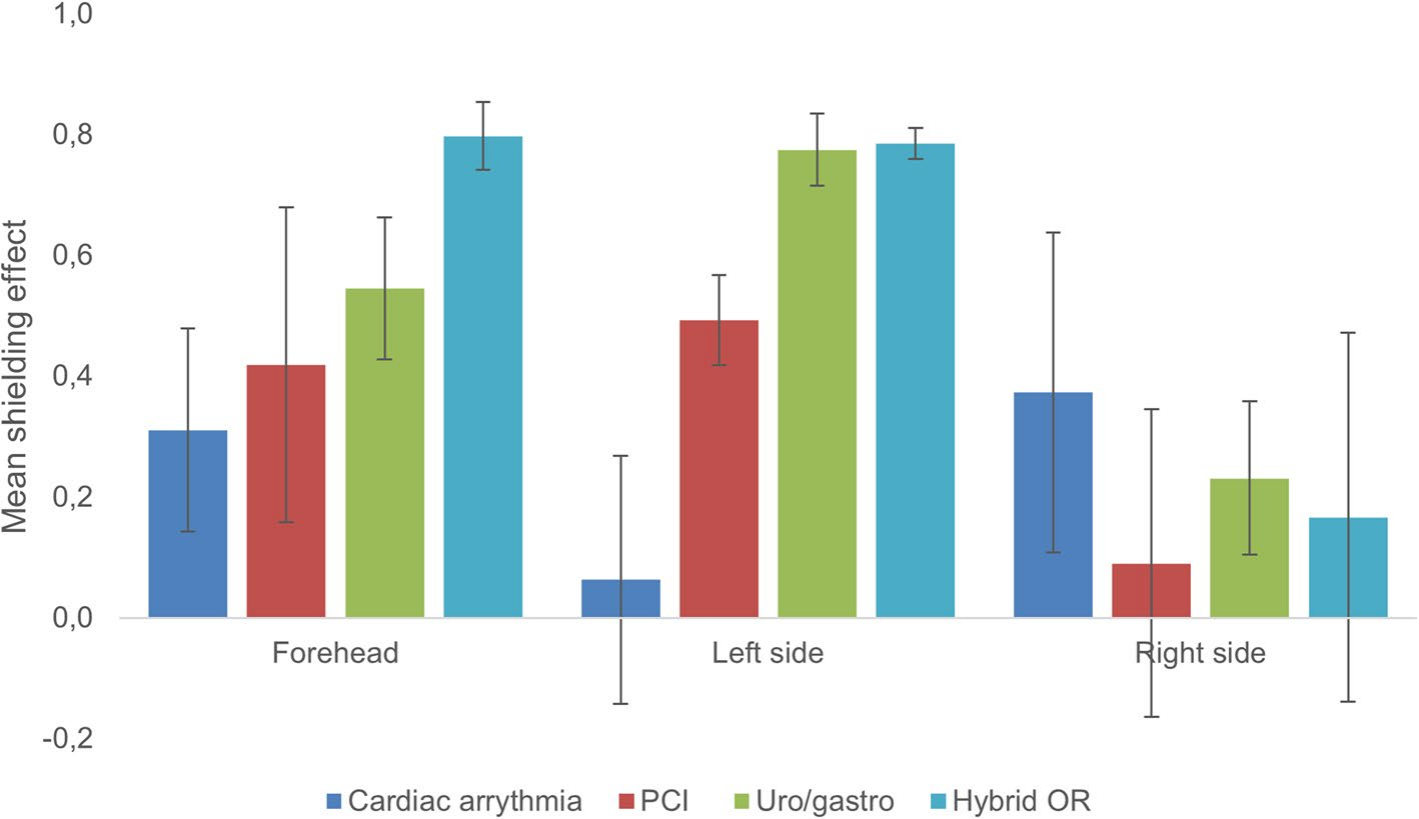
The mean shielding effect, RSE, for the forehead, left and right side of the head in various clinical environments

Using the head protector during radiologically guided interventions, the TLD-signal varied according to the nature of the procedures and the position of the operator relative to the irradiated area. All participants, except five operators performing cardiac electrophysiological interventions, had higher TLD signals on the left side of the head, compared to the right side. The mean shielding effect from the forehead (measuring points 1–2), the left side (measuring points 3–10) and the right side (measuring points 12–15) are presented in Fig. 6. The mean shielding effect for the head protector was 0.4 (SD 0.3; range -0.4 to 0.9). The negative data indicate that the inside TLD-signal was higher than the outside of the head protector. One Hybrid OR operator experienced a reduction from 1100 µSv to 190 µSv, whereas one Percutaneous Coronary Intervention (PCI) operator experienced a reduction from 100 µSv to 33 µSv for the same measuring points, 7 and 8 (left side).

## Discussion

There are several ways to protect medical practitioners during x-ray guided procedures including shields and maximizing distance between the interventionist and the patient. As indicated in this study, the ceiling mounted lead glass shield was the most effective shielding, R_SE_ > 0.6 in all 224 measure-points, reducing exposure of the interventionist’s head with a mean shielding effect of 97%, all data not shown. This is consistent with Meader et al. who found a reduction in eye lens dose by a factor of 19 when using a lead shield, and Batlivala et al. concluded that a lead glass shield mounted on the ceiling provided maximum protection [14, 15]. Effective protection requires that the operator remains behind the shield for the duration of the x-ray guided procedure [16].

The capability of a radiation shield to provide complete protection of a physician’s radiation sensitive body parts may vary according to the clinical situation. The design and position of ceiling mounted lead shields and the knowledge of the direction of the scattered radiation is necessary to ensure optimal protection [12]. Madder et al. for example showed a two-thirds reduction in radiation exposure, for nurses and technicians, when ceiling mounted lead shields were used in the clinic [16]. It has also been shown that the radiation dose reduction factor differs according to the design of lead eyeglasses, depending on the extent of eye coverage and their ability to attenuate the direction of the scattered radiation, which is critical for the dose that reaches the lens of the eye [3, 4, 17–19].

In environments such as hybrid OR, where the levels of scattered radiation can be high, and where a complete protection of a physician is difficult to achieve, the application of a radiation shield with at least 50% dose reduction might provide a substantial reduction in exposure. Similar to the practical use of a DRF of 2 proposed for protective eyewear, this study presents the shielding effect ratio (R_SE_) as green i.e. when R_SE_ > 0.5. This does not necessarily indicate that a 50% reduction is good enough. The radiation protection must always be optimized individually in the clinical situation.

Depending on the position of the operator’s head with respect to the radiation source, the head protector can protect against radiation that encounters its outer surface, in a similar manner to a conventional radiation protective apron, by attenuating radiation and preventing it from entering. However, during x-ray guided intervention the radiation hits the operator’s head at an oblique angle from below, entering through the neck, chin, and face. The extended thyroid protector has been designed to protect against this scattered radiation coming from below. A ceiling mounted radiation protection is, if properly used and positioned, an effective protector of scattered radiation. In situations where a ceiling mounted shield cannot or is not maneuvered into a blocking position, the combination of the head protector and the extended thyroid protector might be a way to reduce brain and head exposure.

A clinical study by Bärenfänger et al. evaluating the head protector used in this study found a superficial mean dose reduction of 75% and 90% for general radiology and neuro radiology procedures respectively [20]. Meanwhile, the clinical evaluation in the present study showed that the head protector reduced the radiation dose to the dosimeters placed on the inside of the protector by about 40%. The differences in the mean dose reduction are probably due to the fact that the current study had more measure-points on both sides of the head protector, lowering the mean dose reduction. However, the present phantom study showed that for the head protector the shielding effect further into the head, towards the center of the skull/brain was minor. The scattered radiation from the patient usually comes obliquely from below and the head protector cannot provide effective protection inside the brain. The phantom study showed that the head protector has the most protective effect superficially close to where it covers the head. In studies evaluating head protection devices similar to the head protector, Fetterly et al. show that head protection at the level of the forehead provides minimal shielding of the head [21, 22]. Kirkwood et al. also found a reduction in superficial dose but a minor dose reduction of the dose to the brain using a head cap [23]. However, head protection devices could be used as a complement when optimal ceiling lead protection positioning is not possible in certain operative situations. In these environments, lead glasses are also recommended [3].

The phantom study showed that the extended thyroid protector prototype (0.5 mm Pb) reduced the radiation dose by up to 90% in the middle sections (slice c) of the head, which is partly due to the novel design. In addition, the extended thyroid protector lowered the radiation dose in the upper section (slice d and e) of the head with a mean shielding effect of 20–30% for the scatter radiation direction in this study. However, the present evaluation of the standard thyroid collar showed no shielding effect in the upper section of the head. In contrast to the standard thyroid collar the extended thyroid protector is not only designed to shield the neck but also the head. In accordance with the present data, Marshall et al. showed the effect of shielding against obliquely arising scattered radiation from below using a lead acrylic face mask that provided an 80% reduction in brain dose, while also protecting eye and thyroid [24]. Although the present study did not include a clinical study with the extended thyroid protector, this was tested clinically by Bärenfänger et al. demonstrating an attenuation effect of the extended thyroid protector resulting in a dose reduction by up to 97% after the barrier [20]. This is consistent with the results in the present phantom study with the extended thyroid protector prototype, reducing the radiation dose by up to 90% in the middle sections of the head. It is important to further evaluate its shielding effect on the thyroid gland itself for different scatter radiation directions to ensure that the thyroid protection is not inferior to that provided by a standard thyroid collar.

The Hp(0.07) calibration of the TLDs for estimation of radiation dose at various depth positions in the phantom introduces an error. However, the calibration for personal dose equivalent Hp(0.07) is of less importance as ratios were used in all results, i.e. R_SE_ = 1-[Hp(0.07)_unshielded_/Hp(0.07)_unshielded_)], and the errors cancel out.

In the head protector and ceiling mounted lead shield studies, all TLD position were used while, due to practical reasons, TLDs were placed more sparsely for the extended thyroid protector. Additional TLDs are not expected to change the schematic illustration of the shielding effect of the thyroid protectors in Fig. 5C and D.

### Limitations

The phantom study has several limitations as neither the angulation of the x-ray tube nor the operator's position varied throughout the experiment. Changing the angulation of incident radiation with respect to the operator changes the radiation exposure situation, affecting the dose distribution inside the operator’s head. In the present study the radiation comes from obliquely below, however, if the radiation entered the operator’s head from more directly, the shielding effect would be a somewhat less for the extended thyroid protector but somewhat higher for the head protector but almost the same for the ceiling mounted shield and the non-shielded set-up, due to the geometry.

The commercially available MindPeace™ with attenuating material of 0.35 mm Pb provides up to 3% less shielding effect in the energy interval 60-110 kV when compared to the extended thyroid protector with attenuating material of 0.5 mm Pb evaluated in this study (unpublished data, Texray AB). It is also important to note that body worn radiation shields may differ in protection effect depending on operator's anatomy in relation to product size and fit.

## Conclusions

For optimal radiation protection of the head, radiation protection devices that protect the entire interventionist’s head against scattered radiation should be used, such as ceiling mounted lead glass shields. However, in clinical practice, optimal positioning of a ceiling mounted lead shield may not always be possible. Operators, particularly during complex cases when radiation protection may be most relevant, cannot always ensure optimal shield placement. Added protection using these novel guards may compliment the effect of the lead shield.

The head protector alone does not provide sufficient protection in clinical situations. A combination of the head protector, the extended thyroid protector and a lead glass shield seem to provide comprehensive radiation protection for the head and neck. As is the case for ceiling mounted lead glass shields, the investigated radiation protection devices will offer most benefit in cases of heavier radiation exposure, such as x-ray guided interventions.

## Data Availability

The data sets can be made available after request to the corresponding author.

## Abbreviations

DAP: Dose Area Product
DRF: Dose Reduction Factor
ICRP: International Commission on Radiological Protection
PCI: Percutaneous Coronary Intervention
PMMA: Polymethyl methacrylate
SID: Source image distance
TLD: Thermoluminescent dosimeters

## References

[1] United Nations. Scientific Committee on the Effects of Atomic Radiation. Sources and effects of ionizing radiation : United Nations Scientific Committee on the Effects of Atomic Radiation : UNSCEAR (2008). report to the General Assembly, with scientific annexes.

[2] Authors on behalf of I, Stewart FA, Akleyev AV, Hauer-Jensen M, Hendry JH, Kleiman NJ, et al. ICRP publication 118: ICRP statement on tissue reactions and early and late effects of radiation in normal tissues and organs--threshold doses for tissue reactions in a radiation protection context. Ann ICRP. 2012;41(1–2):1–322. 10.1016/j.icrp.2012.02.001 22925378

[3] Martin CJ, Magee JS, Sandblom V, Almen A, Lundh C (2015). Eye dosimetry and protective eyewear for interventional clinicians. Radiat Prot Dosimetry.

[4] Magee JS, Martin CJ, Sandblom V, Carter MJ, Almen A, Cederblad A (2014). Derivation and application of dose reduction factors for protective eyewear worn in interventional radiology and cardiology. J Radiol Prot.

[5] The 2007 Recommendations of the International Commission on Radiological Protection. ICRP publication 103 (2007). Ann ICRP.

[6] Little MP, Azizova TV, Bazyka D, Bouffler SD, Cardis E, Chekin S (2012). Systematic review and meta-analysis of circulatory disease from exposure to low-level ionizing radiation and estimates of potential population mortality risks. Environ Health Perspect.

[7] Hendry JH (2015). Threshold doses and circulatory disease risks. Ann ICRP.

[8] Roguin A, Goldstein J, Bar O, Goldstein JA (2013). Brain and neck tumors among physicians performing interventional procedures. Am J Cardiol.

[9] Reeves RR, Ang L, Bahadorani J, Naghi J, Dominguez A, Palakodeti V (2015). Invasive cardiologists are exposed to greater left sided cranial radiation: The BRAIN Study (Brain Radiation Exposure and Attenuation During Invasive Cardiology Procedures). JACC Cardiovasc Interv.

[10] Roguin A, Nolan J (2021). Radiation protection in the cardiac catheterisation lab: best practice. Heart.

[11] Miller DL, Vano E, Bartal G, Balter S, Dixon R, Padovani R (2010). Occupational radiation protection in interventional radiology: a joint guideline of the Cardiovascular and Interventional Radiology Society of Europe and the Society of Interventional Radiology. Cardiovasc Intervent Radiol.

[12] Lopez PO, Dauer LT, Loose R, Martin CJ, Miller DL, Vano E (2018). ICRP Publication 139: occupational radiological protection in interventional procedures. Ann ICRP.

[13] Thermo Fisher Scientific. Thermo Scientific Harshaw TLD Materials and Dosimeters. In: Scientific TF, editor.: Thermo Fisher Scientific 2016.

[14] Maeder M, Brunner-La Rocca HP, Wolber T, Ammann P, Roelli H, Rohner F (2006). Impact of a lead glass screen on scatter radiation to eyes and hands in interventional cardiologists. Catheter Cardio Inte.

[15] Batlivala SP, Magill D, Felice MA, Jones V, Dori Y, Gillespie MJ (2016). The effect of radiation shields on operator exposure during congenital cardiac catheterisation. Radiat Prot Dosim.

[16] Madder RD, LaCombe A, VanOosterhout S, Mulder A, Elmore M, Parker JL (2018). Radiation exposure among scrub technologists and nurse circulators during cardiac catheterization: the impact of accessory lead shields. JACC Cardiovasc Interv.

[17] Principi S, Farah J, Ferrari P, Carinou E, Clairand I, Ginjaume M (2016). The influence of operator position, height and body orientation on eye lens dose in interventional radiology and cardiology: Monte Carlo simulations versus realistic clinical measurements. Phys Med.

[18] Thornton RH, Dauer LT, Altamirano JP, Alvarado KJ, St Germain J, Solomon SB (2010). Comparing strategies for operator eye protection in the interventional radiology suite. J Vasc Interv Radiol.

[19] Honorio da Silva E, Martin CJ, Vanhavere F, Buls N (2020). A study of the underestimation of eye lens dose with current eye dosemeters for interventional clinicians wearing lead glasses. J Radiol Prot.

[20] Barenfanger F, Walbersloh J, El Mouden R, Goerg F, Block A, Rohde S (2022). Clinical evaluation of a novel head protection system for interventional radiologists. Eur J Radiol.

[21] Fetterly K, Schueler B, Grams M, Sturchio G, Bell M, Gulati R (2017). Head and neck radiation dose and radiation safety for interventional physicians. JACC Cardiovasc Interv.

[22] Fetterly KA, Schueler BA, Grams MP, Sturchio GM (2017). Estimating head and neck tissue dose from x-ray scatter to physicians performing x-ray guided cardiovascular procedures: a phantom study. J Radiol Prot.

[23] Kirkwood ML, Arbique G, Guild J, Xi Y, Zeng KT, Rectenwald J (2017). Radiation Brain Dose to Vascular Surgeons During Fluoroscopically Guided Interventions Is Not Effectively Reduced by Wearing Lead Equivalent Surgical Caps. J Vasc Surg.

[24] Marshall NW, Faulkner K, Clarke P (1992). An investigation into the effect of protective devices on the dose to radiosensitive organs in the head and neck. Brit J Radiol.

